# Association between remnant cholesterol inflammation Index and in-hospital New-onset atrial fibrillation in patients with ST-segment elevation myocardial infarction

**DOI:** 10.3389/fcvm.2026.1881907

**Published:** 2026-06-25

**Authors:** Yamin Xu, Xiabing Hu, Yun Qian, Hong Ding, Zhen Li, Xiuyu Ma, Pingzhen Wu

**Affiliations:** 1 Department of Cardiovascular Medicine, Taizhou Hospital of Traditional Chinese Medicine, Taizhou, China; 2 Department of Cardiovascular Medicine, Binhai County People’s Hospital, Binhai, China; 3 Department of Cardiovascular Medicine, Taixing People’s Hospital Affiliated to Yangzhou University, Taixing, China

**Keywords:** new-onset atrial fibrillation, percutaneous coronary intervention, remnant cholesterol inflammatory index, risk stratification, ST-segment elevation myocardial infarction

## Abstract

**Background:**

New-onset atrial fibrillation (NOAF) is a common in-hospital complication among patients with acute ST-segment elevation myocardial infarction (STEMI) and is closely associated with adverse outcomes. The remnant cholesterol inflammatory index (RCII), a novel composite marker integrating lipid metabolism abnormalities and systemic inflammation, has not been well studied in relation to NOAF in STEMI patients. This study aimed to investigate the association between RCII and the occurrence of in-hospital NOAF in patients with STEMI.

**Methods:**

This single-center retrospective observational study consecutively enrolled patients with first-time acute STEMI undergoing emergency percutaneous coronary intervention (PCI) from January 2022 and December 2025. RCII was defined as the product of remnant cholesterol (RC) and high-sensitivity C-reactive protein (hs-CRP).

**Results:**

A total of 945 STEMI patients were included, of whom 79 (8.36%) developed NOAF. Patients with NOAF had significantly higher levels of RCII, RC, and hs-CRP compared with those without NOAF. Multivariable logistic regression analysis demonstrated that hs-CRP (OR = 2.96, 95% CI: 1.72–5.08, *P* < 0.001), RCII (OR = 4.71, 95% CI: 2.86–7.78, *P* < 0.001), and RC (OR = 2.78, 95% CI: 1.88–4.10, *P* < 0.001) were each independently associated with the occurrence of NOAF in separate multivariable models. Restricted cubic spline analysis indicated a significant nonlinear dose–response relationship between RCII and NOAF risk (*P* for overall < 0.001, *P* for nonlinear < 0.05). ROC curve analysis showed that RCII identified NOAF with an area under the curve of 0.756 (95% CI: 0.698–0.814), outperforming RC alone (*Z* = 3.105, *P* = 0.002) or hs-CRP alone (*Z* = 2.805, *P* = 0.005).

**Conclusions:**

Higher RCII was independently associated with an increased risk of in-hospital NOAF and showed moderate discriminatory ability. RCII may serve as a simple, cost-effective, and efficient early risk stratification tool for identifying patients at high risk of NOAF.

## 1 Introduction

Acute ST-elevation myocardial infarction (STEMI) is one of the most critical and life-threatening clinical manifestations of coronary artery disease (1). During the acute phase, patients with STEMI are exposed to intense systemic stress, characterized by excessive sympathetic activation, hemodynamic instability, extensive myocardial necrosis, and a pronounced inflammatory response (2). Within this complex pathophysiological setting, cardiac arrhythmias frequently occur, among which new-onset atrial fibrillation (NOAF) is particularly common and associated with adverse outcomes. Previous studies have reported that the incidence of NOAF during hospitalization ranges from approximately 6% to 21% in STEMI patients undergoing early reperfusion therapy (3–5). Substantial evidence from large cohort studies and meta-analyses has consistently demonstrated that NOAF is an independent predictor of in-hospital and long-term mortality, heart failure, stroke, and other major adverse cardiovascular events in patients with STEMI (6, 7). Therefore, early identification of patients at high risk for NOAF during hospitalization is essential for optimizing monitoring strategies and improving clinical outcomes.

Remnant cholesterol (RC), an atherogenic lipid fraction that has attracted increasing attention in recent years, refers to the cholesterol content carried by triglyceride-rich lipoproteins and their remnants (8). Notably, RC exhibits more potent pro-inflammatory properties, activating the monocyte–macrophage system and amplifying inflammatory responses. Epidemiological studies and Mendelian randomization analyses have established an independent and causal association between elevated RC levels and an increased risk of incident atrial fibrillation (9–11). Mechanistically, RC may contribute to atrial fibrillation by inducing inflammation, oxidative stress, and endothelial dysfunction, thereby promoting structural and electrical remodeling of the atria (12, 13). Meanwhile, C-reactive protein (CRP), a well-established biomarker of systemic inflammation, has also been repeatedly shown to be closely associated with the occurrence of NOAF (14–16).

Given the synergistic roles of lipid abnormalities and inflammation in cardiovascular events, the remnant cholesterol inflammation index (RCII), defined as the product of RC and high-sensitivity CRP (hs-CRP), has been proposed as an integrated risk assessment marker (17). Theoretically, RCII may more accurately reflect the true risk of patients simultaneously exposed to high lipid and inflammatory burdens. Emerging evidence suggests that RCII outperforms individual lipid or inflammatory markers in predicting major adverse cardiovascular events in both the general population and patients with coronary artery disease (17–20). However, the relationship between RC, RCII, and the risk of NOAF during hospitalization in STEMI—a particularly high-risk population—remains insufficiently explored. Therefore, the present study aims to investigate the association between RCII and the occurrence of NOAF in patients hospitalized with STEMI, and to evaluate its potential value as a simple, reliable, and cost-effective risk stratification tool, providing evidence to support early identification and individualized prevention and management strategies for NOAF in STEMI patients.

## 2 Methods

### 2.1 Study design and population

**Figure 1:**
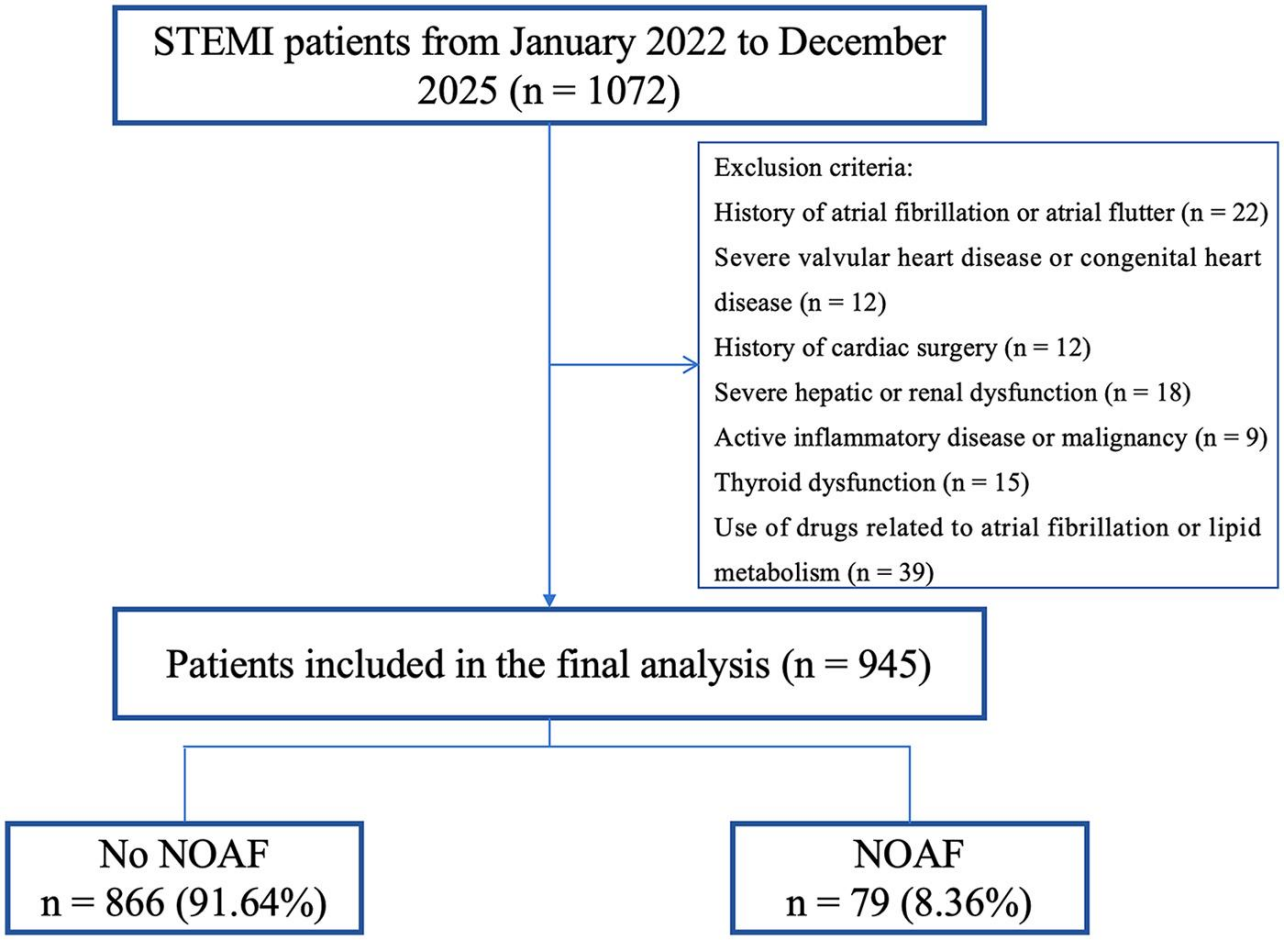
Study flowchart. NOAF, new-onset atrial fibrillation; STEMI, ST-segment elevation myocardial infarction.

This study was designed as a single-center, observational, retrospective cohort study. Consecutive patients who were hospitalized in the Taizhou Hospital of Traditional Chinese Medicine from January 2022 to December 2025, with a first-time diagnosis of acute STEMI, were screened for inclusion. All enrolled patients underwent emergency percutaneous coronary intervention (PCI). Inclusion criteria were as follows: (1) age ≥18 years; (2) diagnosis of STEMI in accordance with the 2017 European Society of Cardiology (ESC) guidelines for the management of ST-elevation myocardial infarction (1); (3) successful emergency PCI within 12 h of symptom onset, defined as post-procedural Thrombolysis in Myocardial Infarction (TIMI) flow grade ≥ 2; (4) continuous electrocardiographic monitoring for at least 72 h after PCI; and (5) complete clinical data available. Exclusion criteria included: (1) documented atrial fibrillation or atrial flutter prior to admission; (2) atrial fibrillation or atrial flutter detected on the admission electrocardiogram; (3) presence of significant valvular heart disease (moderate or severe) or congenital heart disease; (4) prior cardiac surgery, including coronary artery bypass grafting or valve replacement; (5) severe hepatic or renal dysfunction, defined as an estimated glomerular filtration rate (eGFR) < 30 mL/min/1.73 m^2^ or the need for dialysis; (6) acute or chronic active infections, autoimmune diseases, malignancies, or other systemic conditions known to substantially affect inflammatory status; (7) thyroid dysfunction; and (8) regular use of medications prior to admission that could significantly influence lipid metabolism or the occurrence of atrial fibrillation. The inclusion and exclusion flowchart is shown in Figure 1. The study protocol complied with the ethical principles of the Declaration of Helsinki and was approved by the Ethics Committee of the Taizhou Hospital of Traditional Chinese Medicine (approval number: 2026-008-01). Given the retrospective nature of the study and the use of anonymized data, the requirement for written informed consent was waived by the ethics committee.

### 2.2 Data collection

Clinical data were extracted from the electronic medical record system. Data collection was independently performed by two investigators who received standardized training. Any discrepancies were resolved by a senior attending cardiologist. Collected data included baseline demographic characteristics (age, sex, height, weight), medical history and comorbidities (smoking status, hypertension, type 2 diabetes mellitus, prior stroke), and admission vital signs and clinical assessments (systolic blood pressure, diastolic blood pressure, heart rate, and Killip classification). Laboratory parameters were obtained from the first fasting blood samples collected after admission, including total cholesterol (TC), triglycerides (TG), low-density lipoprotein cholesterol (LDL-C), high-density lipoprotein cholesterol (HDL-C), and hs-CRP. Blood samples for lipid profile measurements were obtained from the first fasting blood sample collected after admission. Importantly, these samples were collected prior to the initiation of statin therapy. LDL-C was calculated using the Friedewald formula. RC was calculated using the following formula: RC (mmol/L) = TC (mmol/L)−HDL-C (mmol/L)−LDL-C (mmol/L). RCII was defined as the product of RC and hs-CRP. Additional laboratory variables included eGFR, N-terminal pro–B-type natriuretic peptide (NT-proBNP), high-sensitivity troponin T (hsTnT), and fasting plasma glucose. Due to skewed distributions, RCII, hs-CRP, NT-proBNP, and hs-TnT were natural log–transformed for regression analyses. Left ventricular ejection fraction (LVEF) was obtained from transthoracic echocardiography performed during hospitalization. The culprit vessel was determined based on coronary angiography and PCI records. Medication use during hospitalization was also recorded, including aspirin, P2Y12 inhibitors, statins, *β*-blockers, angiotensin-converting enzyme inhibitors (ACEI), and/or angiotensin II receptor blockers (ARB). NOAF was defined as atrial fibrillation episodes lasting longer than 30 s, characterized by the absence of distinct P waves and an absolutely irregular R–R interval, detected by any 12-lead electrocardiogram or continuous cardiac monitoring during hospitalization. All suspected NOAF events were independently adjudicated by two experienced cardiologists.

### 2.3 Statistical analysis

All statistical analyses were performed using R software (version 4.3.1; R Core Team, Vienna, Austria) and SPSS software (version 26.0; IBM Corp., Armonk, NY, USA). Patients were categorized into two groups according to the occurrence of NOAF during hospitalization. Continuous variables were assessed for normality using the Shapiro–Wilk test. Normally distributed variables are presented as mean ± standard deviation (SD) and were compared using the independent samples *t* test. Non-normally distributed variables are expressed as median with interquartile range [M (Q1, Q3)] and were compared using the Mann–Whitney U test. Categorical variables are presented as counts and percentages [n (%)] and were compared using the chi-square test or Fisher's exact test, as appropriate. Univariable logistic regression analyses were conducted to identify potential factors associated with NOAF. Variables with clinical relevance or those with a *P* value < 0.05 in univariable analyses were subsequently included in multivariable logistic regression models to identify independent factors of NOAF. To assess potential model overfitting and model stability, the events-per-variable ratio was calculated, and bootstrap internal validation with 1000 resamples was performed for the multivariable logistic regression models. The events-per-variable ratio was approximately 9.9 and bootstrap internal validation showed generally stable results. Restricted cubic spline (RCS) analyses were performed to explore potential nonlinear associations between RCII and the risk of NOAF, with adjustment for relevant covariates. Receiver operating characteristic (ROC) curve analysis was used to evaluate the discriminatory performance of RCII for NOAF, the optimal cutoff values in ROC analysis were determined using the Youden index. Comparisons of the area under the curve (AUC) between RCII, RC, and hs-CRP were performed using the DeLong test. Net reclassification improvement (NRI) and integrated discrimination improvement (IDI) were calculated to evaluate the incremental value of adding RCII to the baseline model for risk stratification. All statistical tests were two-sided, and a *P* value < 0.05 was considered statistically significant.

## 2 Results

### 3.1 Baseline characteristics

A total of 945 patients with STEMI were enrolled in this study, among whom 79 patients (8.36%) developed NOAF. The baseline characteristics of the study population are summarized in Table 1. Compared with patients without NOAF, those in the NOAF group were significantly older, had lower systolic blood pressure, and exhibited markedly reduced LVEF (*P* < 0.05). Regarding culprit vessel distribution, right coronary artery (RCA) lesions were more frequently observed in patients with NOAF, whereas left anterior descending artery (LAD) lesions were less common (*P* < 0.05). Moreover, the proportion of patients with Killip class >1 was significantly higher in the NOAF group (*P* < 0.001).

**Table 1:** Patient characteristics in patients with STEMI.

Variables	Total (n = 945)	No NOAF (*n* = 866)	NOAF (*n* = 79)	*P*
Age, years	65.49 ± 12.97	64.81 ± 12.99	72.99 ± 10.15	<0.001
BMI, kg/m^2^	24.73 ± 3.59	24.72 ± 3.43	24.82 ± 5.06	0.872
Heart rate, bpm	78.18 ± 13.87	77.97 ± 13.71	80.47 ± 15.36	0.126
SBP, mmHg	129.98 ± 24.26	130.54 ± 23.77	123.85 ± 28.63	0.047
DBP, mmHg	79.76 ± 31.87	80.10 ± 32.93	76.08 ± 15.83	0.283
LVEF, %	51.80 ± 7.27	52.24 ± 6.85	46.97 ± 9.66	<0.001
TC, mmol/L	4.35 ± 1.07	4.33 ± 1.07	4.56 ± 1.08	0.070
Triglycerides, mmol/L	1.48 ± 1.18	1.46 ± 1.18	1.67 ± 1.20	0.133
LDL, mmol/L	2.66 ± 0.88	2.67 ± 0.88	2.51 ± 0.88	0.117
HDL, mmol/L	1.03 ± 0.27	1.03 ± 0.28	0.99 ± 0.26	0.166
hs-TnT, ng/mL	1661.0 (452.7, 4421.0)	1555.0 (422.7, 4264.5)	3102.0 (811.1, 5916.0)	0.003
NT-pro BNP, pg/mL	2013.7 (890.7, 4742.8)	1840.3 (831.4, 4150.4)	5054.6 (3105.2, 10433.5)	<0.001
hs-CRP, mg/L	23.00 (7.90, 64.60)	20.95 (7.30, 59.90)	64.60 (26.80, 120.70)	<0.001
RCII	12.60 (3.92, 35.40)	11.55 (3.58, 31.80)	53.26 (17.89, 117.99)	<0.001
RC	0.55 (0.38, 0.77)	0.54 (0.38, 0.76)	0.72 (0.48, 1.29)	<0.001
Male, n (%)	689 (72.91)	632 (72.98)	57 (72.15)	0.874
LAD, n (%)	478 (50.58)	449 (51.85)	29 (36.71)	0.010
LCX, n (%)	132 (13.97)	123 (14.20)	9 (11.39)	0.490
RCA, n (%)	300 (31.75)	261 (30.14)	39 (49.37)	<0.001
Others, n (%)	35 (3.70)	33 (3.81)	2 (2.53)	0.791
Hypertension, n (%)	465 (49.21)	428 (49.42)	37 (46.84)	0.660
Diabetes, n (%)	238 (25.19)	214 (24.71)	24 (30.38)	0.267
Stroke, n (%)	138 (14.60)	127 (14.67)	11 (13.92)	0.858
Current smoker, n (%)	372 (39.37)	346 (39.95)	26 (32.91)	0.220
Killip >1, n (%)	169 (17.88)	139 (16.05)	30 (37.97)	<0.001
Aspirin, n (%)	863 (91.32)	787 (90.88)	76 (96.20)	0.108
P2Y_12_ inhibitors, n (%)	904 (95.66)	825 (95.27)	79 (100.00)	0.091
*β*-blockers, n (%)	738 (78.10)	676 (78.06)	62 (78.48)	0.931
Statins, n (%)	888 (93.97)	811 (93.65)	77 (97.47)	0.263
ACEI or ARB, n (%)	503 (53.23)	459 (53.00)	44 (55.70)	0.646

NOAF, new-onset atrial fibrillation; STEMI, ST-segment elevation myocardial infarction; LAD, left anterior descending artery; LCX, left circumflex artery; RCA, right coronary artery; ACEI, angiotensin-converting-enzyme inhibitor; ARB, angiotensin II receptor blocker; HDL, high-density lipoprotein cholesterol; LDL, low-density lipoprotein cholesterol; hs-CRP, high sensitivity C-reactive protein; hs-TnT, high sensitivity TnT; NT-proBNP, N-terminal pro-B-type natriuretic peptide; LVEF, left ventricular ejection fraction; BMI, body mass index; SBP, systolic blood pressure; DBP, diastolic blood pressure; TC, total cholesterol; RC, remnant cholesterol; RCII, remnant cholesterol inflammation index.

Regarding biomarkers, patients in the NOAF group showed significantly higher levels of hs-TnT, and NT-proBNP (*P* < 0.05). In addition, hs-CRP [64.60 (26.80–120.70) vs 20.95 (7.30–59.90), *P* < 0.001], RCII [53.26 (17.89–117.99) vs 11.55 (3.58–31.80), *P* < 0.001] and RC levels [0.72 (0.48–1.29) mmol/L vs 0.54 (0.38–0.76) mmol/L, *P* < 0.001] were significantly higher in the NOAF group (Table 1).

### 3.2 Association between RCII and NOAF in STEMI patients

**Figure 2:**
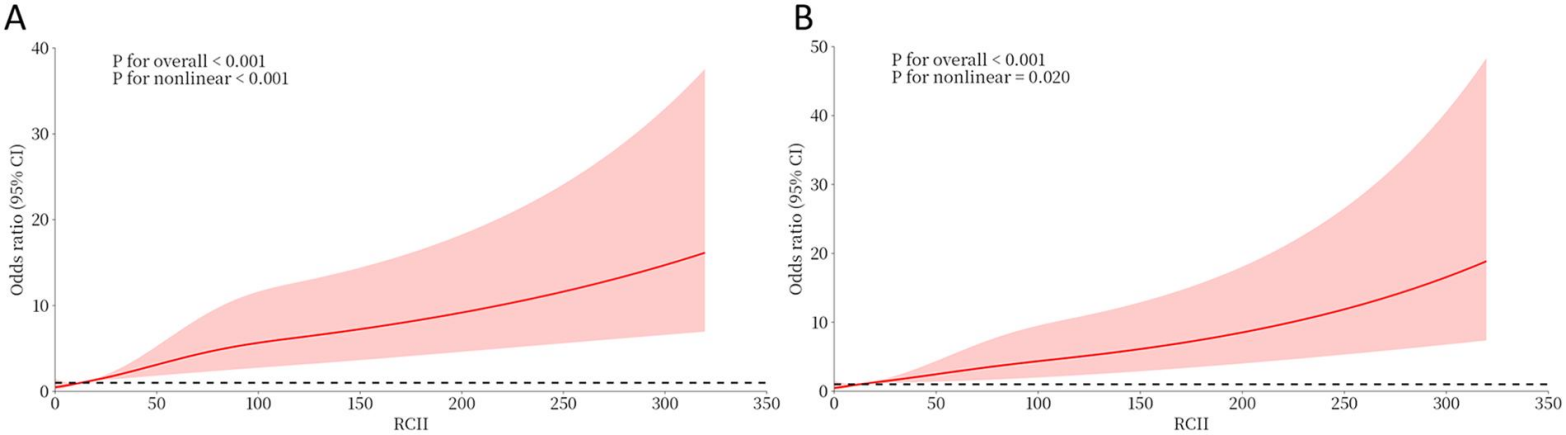
Dose-response relationship in patients with STEMI. **(A)** Unadjusted dose-response relationship between RCII and NOAF. **(B)** Adjusted dose-response relationship between RCII and NOAF, the model was adjusted for age, systolic blood pressure, Killip class, right coronary artery, high sensitivity TnT, N-terminal pro-B-type natriuretic peptide, and left ventricular ejection fraction. The median RCII value was used as the reference. Four knots were placed at the 5th, 35th, 65th, and 95th percentiles of RCII. The solid line indicates the adjusted OR, and the shaded area indicates the 95% CI. NOAF, new-onset atrial fibrillation; STEMI, ST-segment elevation myocardial infarction; RCII, remnant cholesterol inflammation index.

Univariate logistic regression analysis (Supplementary Table 1) demonstrated that age, Killip class, hs-TnT, NT-proBNP, LVEF, RCA, LAD, systolic blood pressure, hs-CRP (OR = 4.20, 95% CI: 2.61–6.75, *P* < 0.001), RC (OR = 2.26, 95% CI: 1.62–3.15, *P* < 0.001), and RCII (OR = 5.26, 95% CI: 3.39–8.17, *P* < 0.001) were associated with the risk of NOAF. Considering the components of RCII, three multivariable logistic regression models were constructed, in which hs-CRP, RCII, and RC were included in Model 1, Model 2, and Model 3, respectively. After adjustment for age, systolic blood pressure, Killip class, RCA, hs-TnT, NT-proBNP, and LVEF, multivariable analysis revealed that hs-CRP (OR = 2.96, 95% CI: 1.72–5.08, *P* < 0.001), RCII (OR = 4.71, 95% CI: 2.86–7.78, *P* < 0.001), and RC (OR = 2.78, 95% CI: 1.88–4.10, *P* < 0.001) were each independently associated with the occurrence of NOAF in separate multivariable models (Table 2). RCS analysis demonstrated a significant nonlinear dose–response relationship between RCII and NOAF risk, both before (*P* for overall <0.001, *P* for nonlinear <0.001) and after (*P* for overall <0.001, *P* for nonlinea*r* = 0.020) adjustment for confounding variables (Figure 2).

**Table 2:** Multivariate regression analysis.

Variables	Model 1		Model 2		Model 3	
	OR (95%CI)	*P*	OR (95%CI)	*P*	OR (95%CI)	*P*
RCA	2.59 (1.56∼4.32)	<0.001	2.71 (1.61∼4.58)	<0.001	2.99 (1.77∼5.04)	<0.001
Age	1.04 (1.02∼1.07)	0.001	1.05 (1.03∼1.08)	<0.001	1.05 (1.02∼1.08)	<0.001
SBP	0.99 (0.98∼1.00)	0.259	0.99 (0.98∼1.00)	0.232	0.99 (0.98∼1.00)	0.243
Log-hs-TnT	1.11 (0.73∼1.70)	0.624	1.01 (0.66∼1.56)	0.952	1.36 (0.88∼2.10)	0.166
Log-NT-proBNP	1.47 (0.75∼2.87)	0.257	1.29 (0.65∼2.57)	0.462	2.38 (1.22∼4.63)	0.011
Killip >1	1.17 (0.65∼2.10)	0.609	1.00 (0.55∼1.83)	0.993	0.98 (0.53∼1.83)	0.948
LVEF	0.94 (0.91∼0.98)	0.001	0.94 (0.91∼0.98)	0.002	0.94 (0.91∼0.98)	0.001
Log-hs-CRP	2.96 (1.72∼5.08)	<0.001	-	-	-	-
Log-RCII	-	-	4.71 (2.86∼7.78)	<0.001	-	-
RC	-	-	-	-	2.78 (1.88∼4.10)	<0.001

RCII, hs-CRP, NT-proBNP, and hs-TnT were log-transformed before regression analyses. The ORs correspond to a one-unit increase in the log-transformed variable. RCA, right coronary artery; hs-CRP, high sensitivity C-reactive protein; hs-TnT, high sensitivity TnT; NT-proBNP, N-terminal pro-B-type natriuretic peptide; LVEF, left ventricular ejection fraction; SBP, systolic blood pressure; RC, remnant cholesterol; RCII, remnant cholesterol inflammation index.

### 3.3 ROC curve analysis

**Figure 3:**
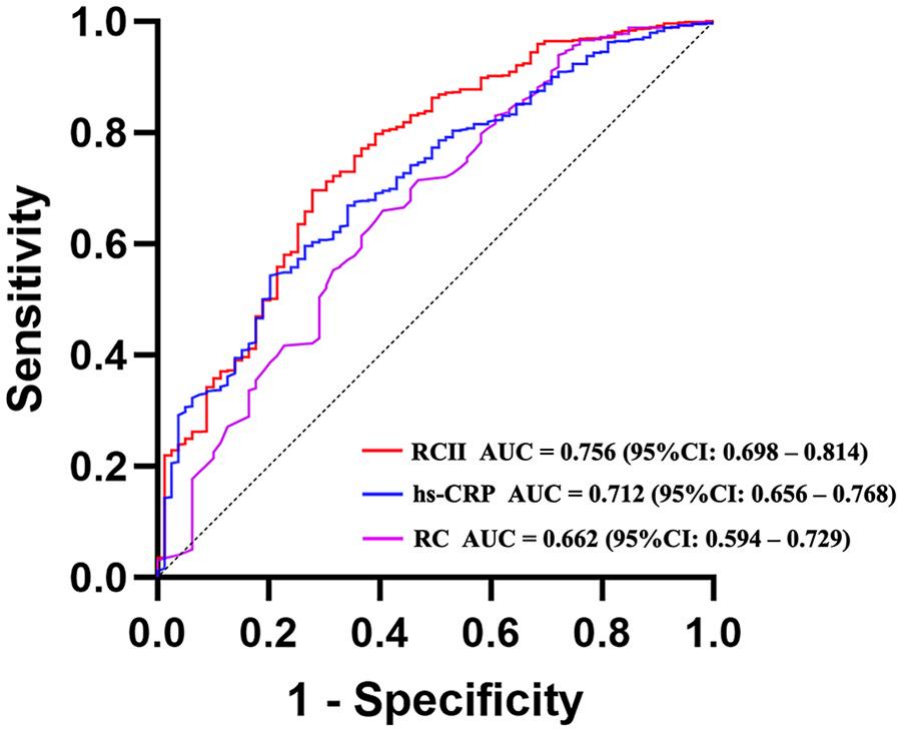
Receiver operating characteristic (ROC) analysis in patients with STEMI. AUC, area under the curve; NOAF, new-onset atrial fibrillation; STEMI, ST-segment elevation myocardial infarction; hs-CRP, high sensitivity C-reactive protein; RC, remnant cholesterol; RCII, remnant cholesterol inflammation index.

ROC curve analysis (Figure 3, Table 3) showed that RCII had an AUC of 0.756 (95% CI: 0.698–0.814, *P* < 0.001) for identifying NOAF, with an optimal cutoff value of 23.79, yielding a sensitivity of 72.2% and a specificity of 69.6%. The AUC for hs-CRP was 0.712 (95% CI: 0.656–0.768, *P* < 0.001), with an optimal cutoff value of 24.05 mg/L, a sensitivity of 91.9%, and a specificity of 54.3%. The AUC for RC was 0.662 (95% CI: 0.594–0.729, *P* < 0.001), with an optimal cutoff value of 0.67, a sensitivity of 82.3%, and a specificity of 66.1%. DeLong's test indicated that RCII exhibited significantly superior performance compared with RC (*Z* = 3.105, *P* = 0.002) or hs-CRP (*Z* = 2.805, *P* = 0.005) alone.

**Table 3:** Receiver operating characteristic analysis.

Variables	AUC	95%CI	Cut-off	Sensitivity	Specificity	*P*
RCII	0.756	0.698∼0.814	23.79	0.722	0.696	<0.001
RC	0.662	0.594∼0.729	0.67	0.823	0.661	<0.001
hs-CRP	0.712	0.656∼0.768	24.05	0.919	0.543	<0.001

hs-CRP, high sensitivity C-reactive protein; RC, remnant cholesterol; RCII, remnant cholesterol inflammation index.

### 3.4 Incremental discriminative and reclassification value of RCII

Based on the multivariable regression analysis, a baseline clinical model incorporating RCA, age, BNP, and LVEF was established. NRI and IDI were subsequently calculated. The addition of RCII significantly improved both the discriminative ability and reclassification performance of the model. Specifically, the IDI was 0.097 (95% CI: 0.053–0.144, *P* < 0.001), and the NRI was 0.722 (95% CI: 0.494–0.943, *P* < 0.001), indicating a significant net reclassification improvement. These findings suggest that RCII improved the discriminative and reclassification performance of the baseline model (Table 4).

**Table 4:** The incremental predictive value of RCII.

Models	NRI		IDI	
	Estimate (95% CI)	*P*	Estimate (95% CI)	*P*
Baseline model	Reference	-	Reference	-
Baseline model + RCII	0.722 (0.494–0.943)	<0.001	0.097 (0.053–0.144)	<0.001

NRI, net reclassification improvement; IDI, integrated discrimination improvement; RCII, remnant cholesterol inflammation index.

## 4 Discussion

In this study, we innovatively focused on a composite biomarker that integrates two key pathological dimensions—lipid metabolism and inflammation—namely the RCII. To our knowledge, limited data are available regarding the association between RCII and in-hospital NOAF in patients with STEMI undergoing emergency PCI. The main findings of our study are as follows: (1) elevated RCII levels were independently associated with an increased risk of NOAF during hospitalization in STEMI patients; and (2) compared with RC or hs-CRP alone, RCII demonstrated superior risk discrimination.

In recent years, RCII has emerged as a novel and clinically meaningful composite indicator, with accumulating evidence supporting its role in cardiovascular diseases (17–20). Analyses based on the U.S. NHANES population have shown that RCII is significantly associated with all-cause mortality and cardiovascular disease risk among individuals with diabetes or prediabetes, highlighting its potential as an integrated marker of lipid abnormalities and low-grade inflammation (19). In another population-based study, both baseline and cumulative RCII exposure were independently associated with an increased risk of incident stroke, with predictive performance superior to that of RC or hs-CRP alone (20). Although remnant cholesterol has been widely reported to be associated with atrial fibrillation, the relationship between RC or RCII, and NOAF in the specific and high-risk population of STEMI patients has remained largely unexplored (12, 13). The central finding of the present study is the strong and independent association between RCII and NOAF. Even after comprehensive adjustment for potential confounders, elevated RCII levels remained significantly associated with a higher risk of NOAF. Furthermore, RCS analyses revealed a clear dose–response relationship between RCII and NOAF incidence. This statistical association may reflect the combined burden of lipid metabolic abnormalities and systemic inflammation, although the underlying mechanisms require further investigation. RC, which is carried by triglyceride-rich lipoprotein remnants, has gained increasing attention for its atherogenic and pro-inflammatory properties (9–11). Unlike LDL-C, which requires oxidative modification to exert pathogenic effects, RC particles can directly penetrate a compromised endothelial barrier and be taken up by macrophages within atrial tissue, thereby triggering a robust local inflammatory cascade (21–23). Compared with LDL-C, RC may exert more pronounced pro-arrhythmic effects through lipid accumulation, inflammation, and atrial lipotoxicity, contributing to electrical remodeling and increased susceptibility to atrial fibrillation (24). In the setting of acute STEMI, systemic stress, ischemia, and hypoxia further impair atrial microvascular endothelial integrity, facilitating RC infiltration and amplification of local inflammation (25, 26). hs-CRP, on the other hand, serves as both an amplifier and a sensitive indicator of systemic inflammation. In patients with STEMI, hs-CRP has been extensively shown to play a crucial role in disease onset and prognosis, including the development of NOAF (14, 27, 28). D’Anna et al. demonstrated in a multicenter study that elevated CRP was independently associated with post-stroke atrial fibrillation, suggesting that the CRP–AF relationship extends beyond myocardial infarction to acute ischemic stroke (29). Importantly, CRP is not merely a passive biomarker; it actively contributes to atrial pathophysiology by downregulating endothelial nitric oxide synthase activity, activating the complement system, and promoting profibrotic signaling pathways (30, 31). These mechanisms collectively lead to microcirculatory dysfunction and atrial fibrosis, thereby providing the structural substrate necessary for reentrant arrhythmias (32, 33). In this context, the observed association between RCII and NOAF in our study appears biologically plausible, although more direct mechanistic evidence from experimental studies is warranted.

Another notable finding of this study is the incremental value of RCII over its individual components in predicting NOAF. Using DeLong tests, we demonstrated that RCII significantly outperformed RC or hs-CRP alone in identifying patients at risk for NOAF. The unique strength of RCII lies in its multiplicative formulation, which may provide integrated information on lipid-related and inflammatory status. RC is a potent pro-inflammatory lipid that upregulates multiple cytokines, including interleukin-6 (IL-6), a key driver of hepatic CRP synthesis, thereby creating a vicious cycle of “elevated RC - intensified inflammation - increased CRP” (34, 35). Conversely, an inflammatory milieu reflected by elevated CRP further exacerbates endothelial injury and permeability, facilitating RC infiltration and pathogenic effects (36). This bidirectional amplification may explain why RCII serves as a more powerful independent correlate of NOAF than either component alone. Clinically, these findings suggest that evaluating lipid abnormalities or inflammatory status in isolation may be insufficient, whereas assessing their interaction may yield more accurate risk stratification.

Although RCA lesions may predispose to NOAF via atrial ischemia, the independent association of RCII with NOAF after adjustment suggests additional systemic mechanisms, such as inflammation and metabolic stress, beyond the site of ischemia (37). In addition, IDI and NRI analyses demonstrated that RCII significantly improved the predictive performance of the baseline NOAF model, including RCA involvement. From a practical perspective, both RC and hs-CRP are routinely measured, standardized laboratory parameters that are inexpensive and readily available in clinical practice. Therefore, RCII represents a simple, cost-effective, and efficient tool for early risk stratification in STEMI patients. Moreover, our findings may provide new insights into preventive and therapeutic strategies for NOAF. For STEMI patients with elevated RCII, treatment goals may need to extend beyond conventional LDL-C–lowering with statins alone, potentially incorporating more aggressive RC-lowering and anti-inflammatory strategies.

### 4.2 Limitations

Several limitations of this study should be acknowledged. First, as a single-center, retrospective observational study, our findings may be subject to selection bias and residual confounding, although extensive multivariable adjustments were performed to mitigate these effects. Second, NOAF detection relied on in-hospital electrocardiography and continuous ECG monitoring. Although all patients received at least 72 h of monitoring after PCI, exact monitoring duration was unavailable. Therefore, brief or asymptomatic episodes may have been missed, and detection bias related to monitoring intensity or hospital stay cannot be excluded. This study also focused only on in-hospital NOAF, and post-discharge AF persistence or recurrence remains unknown. Third, the relatively modest sample size limits the generalizability of our findings, which should be validated in larger, multicenter, prospective cohorts. Although the events-per-variable ratio was approximately 9.9 and bootstrap internal validation showed generally stable results, potential model overfitting cannot be completely excluded because of the limited number of NOAF events. Fourth, LDL-C was calculated using the Friedewald formula, which may be less accurate in patients with elevated triglyceride levels and could potentially affect the estimation of RC. Fifth, although the non-linear relationship between RCII and NOAF suggests a potential threshold effect, the identification of a definitive biological threshold requires further validation. Given the observational nature of this study, caution is warranted in interpreting such thresholds. Finally, hs-CRP and lipid profiles were measured using the first fasting samples after admission, but detailed timing relative to PCI and NOAF onset was unavailable. Thus, RCII may partly reflect acute myocardial injury, reperfusion-related inflammation, or systemic stress, and temporal ambiguity cannot be excluded. Given the retrospective design, causality cannot be established. Left atrial diameter and volume were also unavailable, which may have caused residual confounding.

## 5 Conclusions

Higher RCII was independently associated with an increased risk of in-hospital NOAF and showed moderate discriminatory ability. RCII may serve as a simple, cost-effective, and efficient early risk stratification tool for identifying patients at high risk of NOAF.

## Data Availability

The raw data supporting the conclusions of this article will be made available by the authors, without undue reservation.
