# Supplementary material for: Association between remnant cholesterol inflammation Index and in-hospital New-onset atrial fibrillation in patients with ST-segment elevation myocardial infarction
Source: Front Cardiovasc Med. 2026 Jun 25;13:1881907. doi: 10.3389/fcvm.2026.1881907 (PMC13345855; doi:10.3389/fcvm.2026.1881907)
Supplement: Supplementary file 1 [file Table1.docx]

**Supplemental Table 1.** Univariate Regression Analysis

| Variables | OR (95%CI) | *P* |
| --- | --- | --- |
| Female | 1.04 (0.62 ~ 1.74) | 0.874 |
| LAD | 0.54 (0.33 ~ 0.87) | 0.011 |
| LCX | 0.78 (0.38 ~ 1.60) | 0.491 |
| RCA | 2.26 (1.42 ~ 3.60) | <.001 |
| Others | 0.66 (0.15 ~ 2.78) | 0.567 |
| Hypertension | 0.90 (0.57 ~ 1.43) | 0.660 |
| Diabetes | 1.33 (0.80 ~ 2.20) | 0.268 |
| Stroke | 0.94 (0.48 ~ 1.83) | 0.858 |
| Current smoker | 0.74 (0.45 ~ 1.20) | 0.221 |
| KILLIP >1 | 3.20 (1.96 ~ 5.22) | <.001 |
| Aspirin | 2.54 (0.78 ~ 8.24) | 0.120 |
| β-blockers | 1.03 (0.59 ~ 1.79) | 0.931 |
| Statins | 2.61 (0.63 ~ 10.90) | 0.188 |
| ACEI or ARB | 1.11 (0.70 ~ 1.77) | 0.646 |
| Age | 1.06 (1.04 ~ 1.08) | <.001 |
| BMI | 1.01 (0.94 ~ 1.07) | 0.824 |
| Heart rate | 1.01 (1.00 ~ 1.03) | 0.126 |
| SBP | 0.99 (0.98 ~ 0.99) | 0.019 |
| DBP | 0.99 (0.97 ~ 1.00) | 0.081 |
| Log-hs-TnT | 1.69 (1.17 ~ 2.44) | 0.005 |
| Log-NT-pro BNP | 4.56 (2.88 ~ 7.21) | <.001 |
| Log-hs-CRP | 4.20 (2.61 ~ 6.75) | <.001 |
| LVEF | 0.91 (0.89 ~ 0.94) | <.001 |
| Log-RCII | 5.26 (3.39 ~ 8.17) | <.001 |
| RC | 2.26 (1.62 ~ 3.15) | <.001 |
| TC | 1.20 (0.98 ~ 1.47) | 0.071 |
| Triglycerides | 1.12 (0.96 ~ 1.30) | 0.141 |
| LDL | 0.80 (0.61 ~ 1.06) | 0.117 |
| HDL | 0.53 (0.22 ~ 1.30) | 0.165 |

RCII, hs-CRP, NT-proBNP, and hs-TnT were log-transformed before regression analyses. The ORs correspond to a one-unit increase in the log-transformed variable. NOAF, new-onset atrial fibrillation; STEMI, ST-segment elevation myocardial infarction; LAD, left anterior descending artery; LCX, left circumflex artery; RCA, right coronary artery; ACEI, angiotensin-converting-enzyme inhibitor; ARB, angiotensin II receptor blocker; HDL, high-density lipoprotein cholesterol; LDL, low-density lipoprotein cholesterol; hs-CRP, high sensitivity C-reactive protein; hs-TnT, high sensitivity TnT; NT-proBNP, N-terminal pro-B-type natriuretic peptide; LVEF, left ventricular ejection fraction; BMI, body mass index; SBP, systolic blood pressure; DBP, diastolic blood pressure; TC, total cholesterol; RC, remnant cholesterol; RCII, remnant cholesterol inflammation index.
